# Model building and beyond

**DOI:** 10.1107/S2059798322009330

**Published:** 2022-10-01

**Authors:** Jon Agirre, Robbie P. Joosten, Alan Roseman

**Affiliations:** a York Structural Biology Laboratory, Department of Chemistry, The University of York, Heslington, York YO10 5DD, United Kingdom; b Department of Biochemistry, Netherlands Cancer Institute, Plesmanlaan 121, Amsterdam 1066 CX, The Netherlands; c University of Manchester, Faculty of Biology, Medicine and Health, Oxford Road, Manchester M13 9PT, United Kingdom

**Keywords:** CCP4 Study Weekend, model building, integrative structural biology

## Abstract

An introduction to the Proceedings of the 2020 CCP4 Study Weekend on model building which are available as a virtual issue at https://journals.iucr.org/special_issues/2020/CCP42020/.

The three main macromolecular structure determination techniques are different enough to hold separate congresses and reunions, but there is one topic that connects them like no other: everyone wants to build the best possible atomic model. The 2020 CCP4 Study Weekend, held 7–9 January 2020 in the East Midlands Conference Centre (Nottingham, UK) while COVID-19 was still an outbreak on the other side of the globe, revisited this topic with integrative spirit.

For the first time ever, the meeting opened with a tag-team talk. Eleanor Dodson and Helen Saibil gave a historic perspective and introduced the particularities of model building in macromolecular X-ray crystallography and electron cryo-microscopy (cryo-EM), respectively. The meeting, cross-discipline by design, continued with a session on automated model building, opened by Tom Terwilliger, who discussed his methods for interpreting and improving cryo-EM maps automatically. The session continued with Isabel Usón, who is taking the mantle from George Sheldrick on the development of *SHELXE* and is adapting the software to trace models with maps calculated from electron diffraction data. Kevin Cowtan closed the session with a provocative talk on competitive versus collaborative science, based on their comparison of model building packages, presented in this special issue.

Once a model is obtained, it is time for real or reciprocal space refinement, hence our next session covered several methods for model correction and improvement. Garib Murshudov compared the particularities of X-ray crystallography and cryo-EM maps, laying the foundation for the next talk by Dorothee Liebschner, who discussed methods for automatic refinement of models in cryo-EM maps. Ana Casañal and Tristan Croll presented the latest improvements in real-space refinement in the *Coot* and *ISOLDE* software respectively, with Hamish Todd taking 3D model building to the next level through an exploration of the possibilities and limitations of virtual reality (VR) hardware for interactive model building.

While building a protein can be done fully automatically in an ever growing number of cases, identifying and building ligands is comparatively still a bottleneck due to the absence of a reference sequence, generally poorer density, the potential for multiple binding modes, or the very human self-deception in cases where there is no binding. Mihaela Atanasova discussed the York methods for carbohydrate model building, refinement, validation and analysis. Antonio Rosato covered metals, their coordination and representation in the MetalPDB database. Robert Nicholls offered solutions to the challenges posed by the building and refinement of covalently attached ligands and modifications, such as enzyme–substrate intermediates or protein glycosylation, respectively. Rachael Skyner presented the *Fragalysis* open source software, which allows the user to closely inspect numerous structures of protein–ligand complexes, all stored in the cloud and represented in a web browser. Dinner and the traditional ceilidh followed, paving the way for one of the last traditional social occasions in two years, as the 2021 and 2022 Study Weekends were remote-only events due to the COVID-19 pandemic.

The second day began with a session on low-resolution model building, led by Agnel Joseph who discussed flexible fitting and other tools in *Flex-EM*. Valeriy Titarenko presented methods for the identification of fragments in low-resolution cryo-EM reconstructions, adapting functions traditionally used in crystallographic molecular replacement. Helen Ginn introduced the *Vagabond* refinement software, where molecules are refined in torsion angular space. Sarah Harris explained how molecular dynamics can help even in projects where large molecular size can be a problem.

The next two sessions covered protocols and tools to use whenever a model is nearing deposition: validation, analysis and representation were brought into the foreground. Wah Chiu presented the results from the EM validation challenge; while most tools for model validation are easily transplantable from X-ray crystallography, certain problems are particular to EM and need dealing with as the average resolution of EM structures keeps improving. John Berrisford gave an overview of the tools and procedures associated with protein structure deposition in the Protein Data Bank in Europe and beyond – John was also a driving force behind the deposition tools in both *CCP4i*2 and CCP4 Cloud interfaces. Jiří Černý discussed methods for the validation of nucleic acid structures which, very much like saccharides in general, are of paramount importance to biological structures but have been left behind in method development in favour of a protein-centric perspective. Rafiga Masmaliyeva presented *toBvalid*, a tool for the analysis and validation of temperature factors. In the next session, but largely under the same topic, Anastassis Perrakis gave an overview of the validation and model improvement services in *PDB-REDO*. Marc Baaden showcased his software for the representation and analysis of macromolecules in VR in another example of gaming technology aiding scientists. David Sehnal concluded the session offering an overview of Mol*, the default molecular webviewer used by the PDB websites.

The meeting concluded with a mesmerizing talk by Jeroen Claus of Phospho Ltd, who demonstrated the application of industry-standard 3D modelling tools to the high-end visual­ization of macromolecular atomic structures.

The cover competition, which ran for the first time in the 2020 Study Weekend, produced a number of high-quality submissions. The winner of the contest, Marco Salamina’s ‘Structural Biology: the lost structure’, is showcased on the cover of both the virtual and printed editions, as well as the graphical abstract for this introduction.

We are grateful to CCP4 and its people for funding, championing and running the meeting. And, more than ever, we would like to acknowledge the work all the speakers put into their well crafted talks and carefully prepared papers for this proceedings issue, which is available as a virtual issue at https://journals.iucr.org/special_issues/2020/CCP42020/, and is now complete after a two-year winter.

